# Supplementary material for: The Safety and Efficacy of 1-Monoeicosapentaenoin Isolated from the Trebouxiophyceae Micractinium on Anti-Wrinkle: A Split-Face Randomized, Double-Blind Placebo-Controlled Clinical Study
Source: J Clin Med. 2023 Jan 11;12(2):587. doi: 10.3390/jcm12020587 (PMC9863355; doi:10.3390/jcm12020587)
Supplement: Supplementary file 1 [file jcm-12-00587-s001.zip › Supplementary Table S1.pdf]

Supplementary Table S1. Skin adverse reactions (n = 24)

Symptom		4W		8W		12W	
		A	B	A	B	A	B
Subjective irritation	Itching	0	0	0	0	0	0
	Prickling	0	0	0	0	0	0
	Tickling	0	0	0	0	0	0
	Burning	0	0	0	0	0	0
	Stinging	0	0	0	0	0	0
	Stiffness	0	0	0	0	0	0
	Tightening	0	0	0	0	0	0
	etc.	0	0	0	0	0	0
Objective Irritation	Erythema	0	0	0	0	0	0
	Edema	0	0	0	0	0	0
	Scale	0	0	0	0	0	0
	Papule	0	0	0	0	0	0
	etc.	0	0	0	0	0	0
Total number of subjects		0	0	0	0	0	0

A; Test group, B; Control group
