# Supplementary figures and images for: The Safety and Efficacy of 1-Monoeicosapentaenoin Isolated from the Trebouxiophyceae Micractinium on Anti-Wrinkle: A Split-Face Randomized, Double-Blind Placebo-Controlled Clinical Study
Source: J Clin Med. 2023 Jan 11;12(2):587. doi: 10.3390/jcm12020587 (PMC9863355; doi:10.3390/jcm12020587)

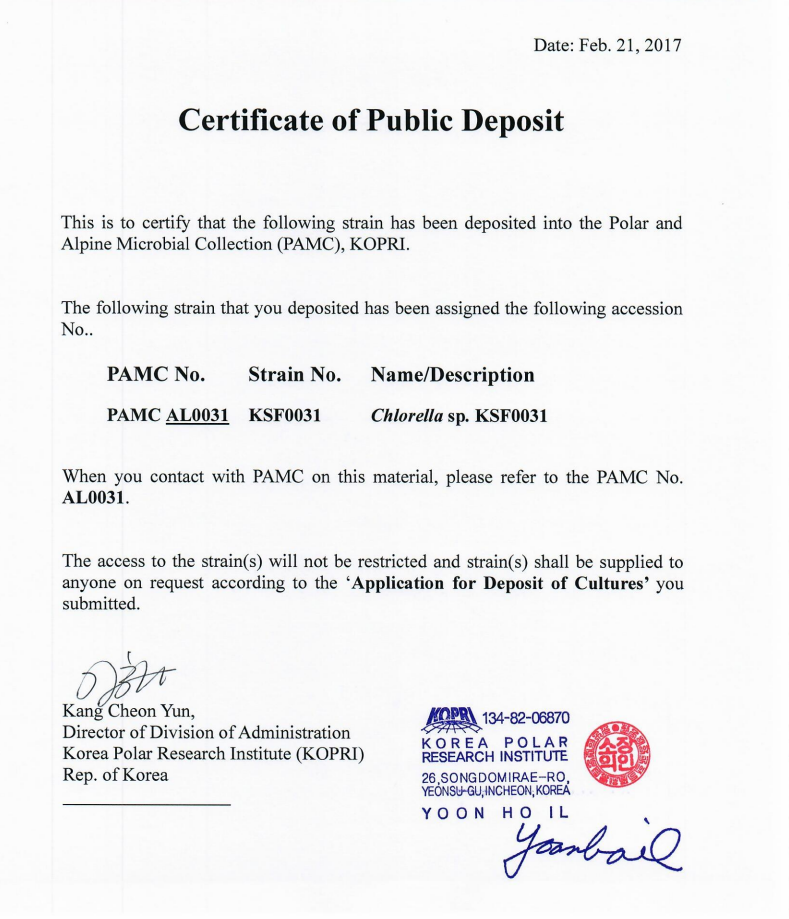

Supplement: Supplementary file 1 [file jcm-12-00587-s001.zip › Supplementary Figure S1.png]
